# The mechanics of the retina: Müller glia role on retinal extracellular matrix and modelling

**DOI:** 10.3389/fmed.2024.1393057

**Published:** 2024-09-04

**Authors:** Laura Prieto-López, Xandra Pereiro, Elena Vecino

**Affiliations:** ^1^Experimental Ophthalmo-Biology Group, Department of Cell Biology and Histology, University of Basque Country UPV/EHU, Leioa, Spain; ^2^Begiker-Ophthalmology Research Group, BioCruces Health Research Institute, Cruces Hospital, Barakaldo, Spain

**Keywords:** extracellular matrix, stiffness, Müller glia, retina, hydrogels

## Abstract

The retina is a highly heterogeneous tissue, both cell-wise but also regarding its extracellular matrix (ECM). The stiffness of the ECM is pivotal in retinal development and maturation and has also been associated with the onset and/or progression of numerous retinal pathologies, such as glaucoma, proliferative vitreoretinopathy (PVR), age-related macular degeneration (AMD), epiretinal membrane (ERM) formation or uveitis. Nonetheless, much remains unknown about the biomechanical milieu of the retina, and specifically the role that Müller glia play as principal mechanosensors and major producers of ECM constituents. So far, new approaches need to be developed to further the knowledge in the field of retinal mechanobiology for ECM-target applications to arise. In this review, we focus on the involvement of Müller glia in shaping and altering the retinal ECM under both physiological and pathological conditions and look into various biomaterial options to more accurately replicate the impact of matrix stiffness *in vitro.*

## Graphical abstract

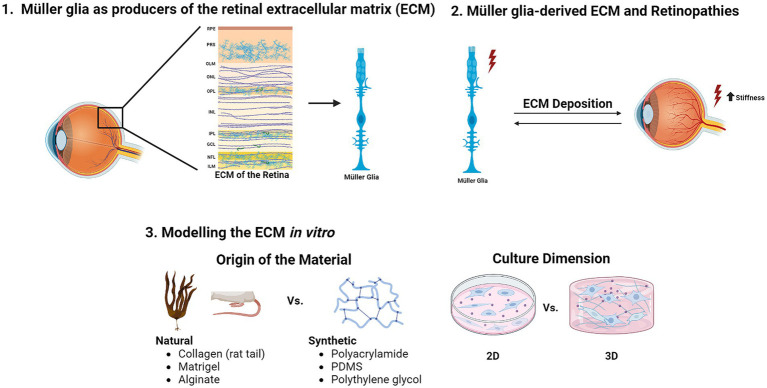
 Visual summary illustrating the key sections discussed in this review. Created with BioRender.com.

## 1 Introduction

Over the last decades, the focus has been put on deciphering how biochemical cues control tissue formation and homeostasis. However, the significance of the physical microenvironment itself has been overlooked. Therefore, by harnessing the unique properties of biomaterials to create *in vitro* models, research is taking a further step in understanding how biomechanical factors influence tissue behaviour, particularly in those subjected to intense mechanical stimuli, such as the retina.

The retina is a tissue belonging to the central nervous system (CNS), responsible for the reception of light stimuli and their transformation into electrical signals (1). Due to its location at the back of the eye, where it is only attached to the eyeball at the *ora serrata* and at the optic nerve head, it is constantly exposed to physical stresses.

The retina endures both static pressures, i.e., the negative pressure pulling on the outer retinal surface exerted by the retinal pigment epithelium (RPE) pumping fluid from the retina to the choroid and the positive pressure on the inner retinal surface created by the tamponade effect of the vitreous body. However, it also suffers a more dynamic pressure; since the vitreous body is attached to both the lens and the retina, it exerts mechanical forces at the retinal surface and at their adhesion sites as the result of every eye movement (2).

Regarding the tissue itself, the retina consists of different layers of intricately shaped cells, extracellular matrix (ECM) and blood vessels, where variations in their composition and distribution suggest a highly mechanically heterogeneous environment (3). For example neurons, including retinal ganglion cells (RGCs), which are the neurons in charge of transmitting the visual information to the brain, while stiff cells themselves require a more compliant environment — i.e. a soft substrate—for their neurites to grow (4, 5). Conversely, Müller glia, the main glial cell type in the retina, despite being softer than RGCs favour a less compliant substrate (3, 5) since it allows them to stretch and be in contact with the different retinal layers. Indeed, Müller glia have been found to be an optimal substrate for RGCs to grow on (6, 7), also promoting neuritogenesis among different species via secretion of neuroprotective factors [for a more comprehensive review of the neuroprotective role of Müller glia see García and Vecino (8) and Vecino et al. (9)].

In this context, the retinal ECM, the acellular part of the tissue on which cells grow, plays a key role in the homeostais of the retina (10). Specifically its rigidity or stiffness (i.e., its capability to resist deformation in response to an applied force) (11) emerges as a determining factor under both retinal physiological and pathologic conditions. In retinal development and homeostasis the ECM rigidity allows the correct organization of the different cell layers and cell differentiation and maturation (12–14). However, in many retinal pathologies, such as diabetic retinopathy (15), age-related macular degeneration (16), proliferative vitreoretinopathy (17) or the formation of epiretinal membranes (18), ECM remodelling and abnormal deposition is involved. Thus, the control of the ECM stiffness plays a key role in regulating the morphology, gene expression, differentiation and overall health of the different retinal cell types (19).

Over the last 20 years, many protein families that respond to mechanical forces (compression, tension, stiffness…) have been discovered. Particularly, several members of the TRP (transient receptor potential) superfamily, first discovered in 1969 (20), were later on found to be mechanosensitive (21). Likewise, in 2010, the Patapoutian group discovered two transmembrane cation channels, Piezo1 and Piezo 2, which were mechanically activated and involved in pressure sensing (22). Focusing on the retina, different studies show that both the tissue itself and adjoining tissues express multiple types of mechanosensitive TRP, particularly TRPV4 and TRPV1 and both Piezo channels (23–26).

Müller glia display long processes and side branches, spanning the entire neural retinal thickness, and thus being in contact with all retinal neuron cell types. Thanks to their privileged location and morphology, not only they provide the necessary tensile strength to maintain tissue integrity (27, 28) but they are also able to sense and respond to even minimal mechanical changes in the retinal structure. Indeed, Müller glia express both pressure/stretch-activated mechanoreceptors TRPV4, Piezo1, and to a lesser extent Piezo 2 (29), which upon activation elicit both a fast response via intracellular calcium signaling and a slow response via changes in protein expression, adapting the environment for their neighbouring neurons (30). Thus, Müller glia are the main retinal mechanosensors and also the major producers of ECM constituents (13, 31). The ECM plays a vital role in Müller glia activation and subsequent gliotic process (32). For this reason, Müller glia should be more closely studied to better understand the mechanical behaviour of the retina.

Although *in vivo* studies ideally would be better to look into the retinal ECM, and indeed several disease animal models have been used to that end (33, 34), in this type of studies there are many variables that difficult the study of a particular mechanical parameter. Thus, biomaterials emerge as a good alternative for *in vitro* models that allow a tight control of the substrate stiffness while more closely recapitulating physiological ECM conditions than traditional cultures.

In this comprehensive review, we aim to deepen our understanding of the retinal physical environment. We will provide context on the distinct characteristics of the retinal ECM and elucidate the impact of matrix stiffening on the onset and progression of common retinal pathologies, focusing on the role played by Müller glia as major mechanosensors and ECM contributors. The characteristics of different biomaterials suitable for *in vitro* modelling of the retinal ECM to further advance our understanding of its biomechanical properties will be explored.

## 2 The ECM of the retina

The ECM forms a complex yet organized network that surrounds the cells and confers structural and mechanical support to tissues. It is a highly dynamic structure in constant change which creates a complex milieu for the cells, regulating cellular homeostasis and signaling both through biochemical signals (e.g., hormones, growth factors and diffusible morphogens) and mechanical cues (e.g., matrix stiffness; tensile, compressive and shear forces; topographical strain) (13, 35, 36).

**Figure 1:**
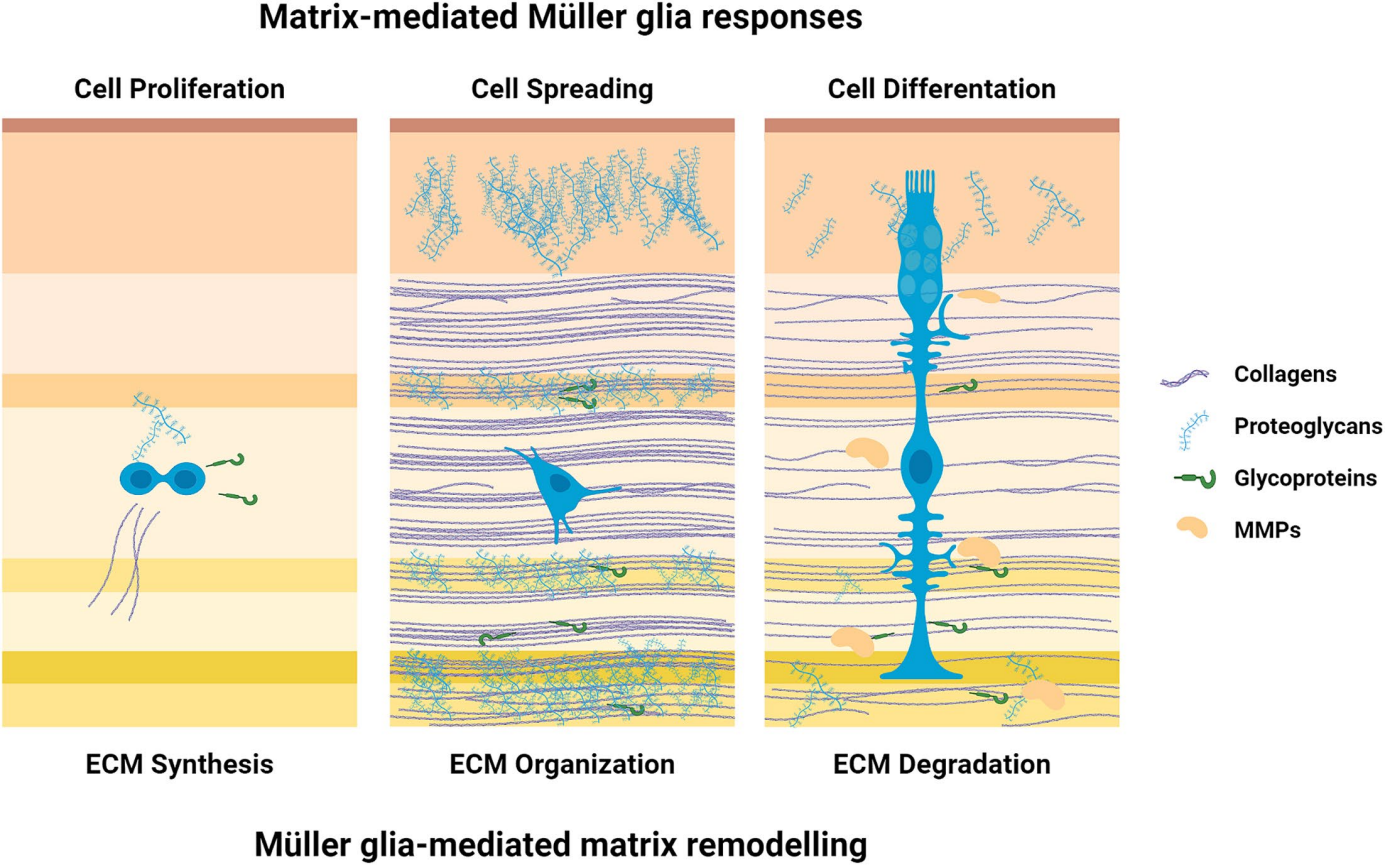
Cell-matrix interactions. There is a two-way communication between the ECM and Müller glia; ECM influences the celular outcome while those same cells organize the ECM. Created with BioRender.com.

The ECM transmits mechanical cues to resident retinal cells and guides their fate by modulating cell growth and differentiation (37). Meanwhile, the cells, primarily Müller glia and astrocytes, can change the composition and macromolecular network structure by secreting matrix components and matrix metalloproteinases (MMPs) or exerting mechanical forces to regulate the arrangement of the ECM (18) (Figure 1).

Müller glia, established as the retina main mechanosensors (30) respond to shifts in the stiffness of the substrate through a mechanical–chemical process that consists in the mechanosensation, mechanotransduction, and downstream mechanoresponses to rigidity stimulus (38). At first, focal adhesions, nanoscaled mechanosensors located on the cell membrane, are formed to link the Müller glia cytoskeleton to the substrate. Then, the rigidity of the microenvironment is sensed and transduced into biochemical signals, triggering changes in cell behaviours, including cell morphology and differentiation (12, 39, 40), and the shuttling of cytoplasmic proteins, such as YAP (yes-associated protein) and TAZ (tafazzin; YAP co-activator), to the nucleus to further regulate cell response has also been linked to Müller glia mechanoresponse (15, 17). Upon increased substrate stiffness, Müller glia activate actin filament extension, leading to cell hypertrophy, and upregulate the production of ECM proteins (41).

### 2.1 Components of the retinal ECM secreted by Müller glia

**Figure 2:**
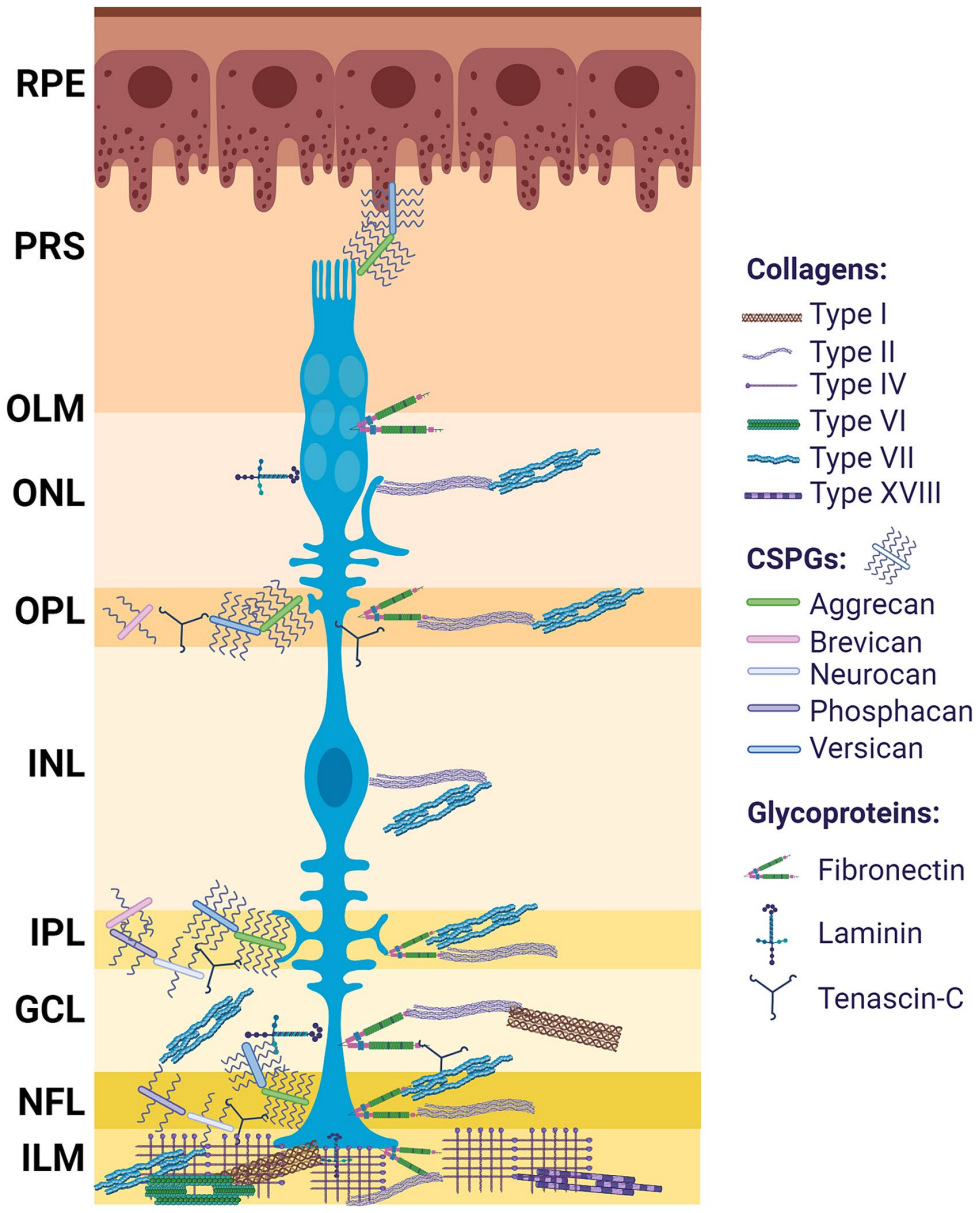
ECM distribution in the retina. Differential distribution of ECM components among the retinal layers; from outermost to innermost: retinal pigment epithelium (RPE), photoreceptor segment (PRS), outer limiting membrane (OLM), outer nuclear layer (ONL), outer plexiform layer (OPL), inner nuclear layer (INL), inner plexiform layer (IPL), ganglion cell layer (GCL), nerve fiber layer (NFL), optic nerve (ON). Müller glia (blue). Created with BioRender.com.

In the retina, ECM components have been mainly associated with basal membranes (BMs), but also with non-BMs (42). The retinal ECM is composed mainly of collagens, proteoglycans and glycoproteins (Figure 2) as seen summarized in Table 1.

**Table 1:** Major ECM components secreted by Müller glia.

ECM component	Producer	Retinal layer	ECM-cell interaction	References
Collagens				
Collagen I	Müller glia Retinal astrocytes	GCL ILM	Direct contact with RGCs in the GCL In contact with Müller glia end-feet forming the ILM Glial scar formation	(44, 46)
Collagen II	Müller glia Hyalocytes	ONL OPL INL IPL GCL NFL ILM	Form the collagen intraretinal network by binding to Müller glia ILM remodelling Retinal blood vessel encapsulation	(47–51)
Collagen IV	Müller glia	ILM	ILM integrity Retinal blood vessel encapsulation RPE anchoring	(31, 47, 53–55)
Collagen VI	Müller glia	ILM	Vitreoretinal attachment	(56)
Collagen VII	Müller glia	ONL OPL INL IPL GCL NFL ILM	Vitreoretinal attachment	(56)
Collagen XVIII	Müller glia	ILM	Vitreoretinal attachment RPE integrity	(56, 57)
Proteoglycans				
Aggrecan	Müller glia Retinal neurons	PRS OPL IPL GCL	Support RGCs and photoreceptors	(61, 62, 64)
Brevican	Müller glia Developing/reactive astrocytes	OPL IPL	Stabilise synapsis Contact with RGC somata Retinal integrity	(61, 66, 67)
Neurocan	Müller glia	IPL NFL	Retinal lamination Inhibitory effect on RGCs neurite outgrowth	(42, 62, 68)
Phosphacan	Müller glia	IPL NFL	Retinal lamination Inhibitory effect on RGCs neurite outgrowth Glial scar formation	(13, 33, 42, 68)
Versican	Müller glia Retinal neurons	PRS OPL IPL GCL	Attachment of the neuroretina to the RPE	(61–63)
Glycoproteins				
Fibronectin	Müller glia Astrocytes	OLM OPL IPL GCL NFL ILM	Maintain cell–matrix adhesion sites Scaffolding of retinal layers Endothelial radial migration during retinal angiogenesis RPE anchoring Müller glia anchoring to the ILM Neurite outgrowth of RGCs	(19, 69–73)
Laminin	Müller glia	ONL GCL ILM	Differentiation and maintenance of the retinal vascular membrane Formation and stability of the ILM and retinal lamination in association with Müller glia Photoreceptor synapsis formation and ONL organization	(53, 73, 76–78, 80)
Tenascin-C	Müller glia Horizontal cells Amacrine cells Astrocytes	OPL IPL NFL	Neurite outgrowth and glial cell differentiation Prevents oligodendrocyte precursors from migrating to the retina During gliosis, contributes to Müller glia ECM deposition, retinal stiffness, photoreceptor degeneration and neuroinflammation Promotes the generation of stable focal adhesions between Müller glia and the ECM Increase sensitivity to FGF2-dependent dedifferentiation of Müller glia *in vitro*	(13, 40, 81–89)

#### 2.1.1 Collagens

Collagens are the main components of the ECM of most soft tissues, including the retina, where they form a fibrillar net in charge of maintaining its structural strength, its attachment to the vitreous and the retinal vasculature (43). The main collagens in the retina secreted by Müller glia are collagen types I-VII and XVIII.

**Collagen I** is the most common fibril-forming collagen in vertebrates. It is both synthesised by retinal astrocytes in direct contact with RGCs and Müller glia (44), thus being an integral part of the ganglion cell layer (GCL) and the inner limiting membrane (ILM), a membrane mostly comprise of Müller glia end-feet that separates the vitreous from the retina. Upregulation of collagen I has been linked as a response to IOP elevation (45) and as a major component of the glial scar in the wound healing process (46).

**Collagen II** has been found mainly around retinal blood vessels, as part of the ECM sheath that encapsulates retinal vasculature (47) but also as isolated deposits in the retina, which have been suggested to intervene in the remodelling of the ILM and the collagen intraretinal network by associating with Müller glia via integrin α1β1 and α2β1 (48, 49). Although in the ILM collagen II is mainly generated by the vitreous-originated hyalocytes (50), Müller glia express the transcription factor Sox9, which directly regulates the gene encoding collagen II (51).

**Collagen IV** is secreted by Müller glia and is the principal ECM component of the ILM of the retina, comprising approximately 60% of its total proteins (45, 52). Due to its structural role, it has been reported to participate in RGCs survival by maintaining ILM integrity, as well as in retinal angiogenesis, where it is part of the collagen network surrounding new vessels (31, 47, 53). It has also been found in the Bruch’s membrane, anchoring the RPE and intervening in its differentiation and wound healing (54, 55).

**Collagens VI, VII,** and **XVIII** are secreted by Müller glia in the ILM of the retina which suggest a role in vitreoretinal attachment due to their matrix anchoring function (56). Collagen VI has also been described in retinal blood vessels and collagen VII is found in most retina layers (NFL, GCL, IPL, INL, OPL, and ONL), especially at the optic nerve head. Furthermore, endostatin, proteolytically derived from Collagen XVIII, has been associated with a protection against neovascularization and maintaining RPE integrity (57).

#### 2.1.2 Proteoglycans

Proteoglycans are composed of a core protein on which long glycosaminoglycan (GAG) chains are attached (58). Depending on which disaccharide conforms said chains, proteoglycans are classified as: hyaluronan (HPGs), chondroitin sulfate (CSPGs), dermatan sulfate (DSPGs), heparan sulfate (HSPGs) and keratan sulfate (KSPGs).

In retinal tissues, CSPGs are secreted primarily from the retinal cells *per se*, and constitute the ECM, whereas other types of proteoglycans including HSPGs, that also predominantly expressed in the retina, remain bound to the cell membrane and do not contribute to the formation of the ECM (42). Especially during retinal development, CSPGs represent major constituents of the ECM, being found in all plexiform layers of the retina and contributing to neural network formation by creating inhibitory boundaries that direct RGC axons. In the adult CNS, CSPGs are typically upregulated in response to injury or neurodegeneration, blocking axonal and cell migration and regeneration as part of the glial scar, created by Müller glia in the retina (59, 60). In the CNS aggrecan, versican, brevican, neurocan and phosphacan are the most common CSPGs (58).

**Versican and aggrecan** are released by both neurons and Müller glia and are located in the inner plexiform layer (IPL) and the GCL of the retina (61), as well as the photoreceptor segment (PRS) and the outer plexiform layer (OPL) (62). Versican plays an important role in the attachment of the neural retina to the RPE (63). The main role of aggrecan is to maintain RGC structure in the IPL and to provide support to photoreceptors (64), which is compromised in gliotic conditions, where Müller glia expression of aggrecan is downregulated (65). Both aggrecan and versican are reported to be more prevalent in ageing and degeneration models, probably due to a higher content of GAG chains compared to other CSPGs that may make up an inhibitory barrier (62).

**Brevican** is secreted by Müller glia and proliferating astrocytes in development and gliosis in the plexiform layers of the retina, where it stabilizes neural synapsis, and in association with RGC somata (61, 66). In physiological conditions its expression is tightly controlled by Müller glia via the miRNA miR-9, which appears to be essential to maintaining retinal integrity. Indeed, in a genetic model where miR-9 regulation was lost, brevican overexpressing Müller glia aggregated, migrated to abnormal locations in the OPL, the outer nuclear layer (ONL), and the IPL and lost cell polarity, leading to a loss of retinal tensile strength and overall retinal disorganization (67).

**Neurocan** and **phosphacan** have been suggested to being involved in the establishment and upkeep of retinal lamination (62). During retinal development, both CSPGs are highly associated with the IPL and nerve fiber layer (NFL) (42), where they display an inhibitory effect on RGCs neurite outgrowth (68). In the adult retina, phosphacan expression is restricted to Müller glia (13), where it has been linked to playing a role in the formation of the glial scar during reactive gliosis as a consequence of RGC damage in a genetic glaucomatous model (33).

#### 2.1.3 Glycoproteins

**Fibronectin** is a glycoprotein, which N-terminal portion contains a self-assembly domain that allow the formation of a fibronectin matrix. As such, fibronectin functions as a regulator of cellular processes, and directs and maintains tissue organization and ECM composition and remodelling. For example, type I, III, and IV collagens depend on fibronectin for their incorporation into the ECM; it has been observed that although integrins α_11_β_1_ and α_2_β_1_ are able to promote fibrillogenesis of type I and type III collagens, a collagen network cannot be formed in the abscence of a preconstituted fibronectin matrix and, more so fibronectin is required to maintain the composition of cell–matrix adhesion sites (19). During retinal development, Müller glia and astrocyte-secreted fibronectin has been found in the interstitial matrix of the retina acting as scaffolding, and its disruption has been linked to the dissolution of retinal layers, neuronal cell death and Müller glia activation (69). Fibronectin has also been shown to be key in endothelial radial migration during mouse retinal angiogenesis (70). However, in the adult retina fibronectin seems to be restricted to a structural component of the RPE and retinal vessels (19), as well as act as anchors of Müller glia end-feet to the ILM (71, 72). Interestingly in *in vitro* studies the fibronectin receptor α5β1 integrin, has been linked to neurite outgrowth of RGCs grown on various ECM substrates as a model for RGC regeneration in the mature retina (73).

**Laminins** are a family of heterotrimeric glycoproteins, composed of one α, one β and one γ chain, which play an important role in multiple biological processes such as adhesion, differentiation, migration, and resistance to apoptosis via cell membrane receptor signaling (74, 75). Members of the laminin family, mainly γ3, α4 and α5 are major components of the retinal vascular basement membrane and play a functional role in its differentiation and maintenance (76). Laminin has been observed in the ganglion cell layer (GCL) and in close association with Müller glia end-feet in the ILM (73, 77). Müller glia is considered the main producer of laminin in the developing rat retina, and specifically the β2 and γ3 laminin chains are key to the formation and stability of the ILM and retinal lamination during development, while their disruption has been linked to Müller glia disorganization, and consequently RGC apoptosis (53, 77). These two laminin chains have also been connected to photoreceptor synapsis formation and the organization of the outer nuclear layer (ONL) (78). Furthermore, it has been observed that laminin stimulates the motility of Müller glia through the activation of its receptor dystroglycan (79). Additionally, in cultures of both mature RGCs and Müller glia, laminin (plus poli-L-lysine) yielded the greatest survival rate in different substrates (73, 80).

**Tenascin-C** (tnc) is a glycoprotein expressed by horizontal and amacrine cells in the plexiform layers of the retina, as well as astrocytes in the optic nerve, exhibiting both adhesive and anti-adhesive properties (13, 81). During development of the CNS, tenascin-C regulates neurite outgrowth and glial cell differentiation (82, 83), and it also acts as a barrier in the optic nerve preventing oligodendrocyte precursors from migrating to the retina (84). However, upon retinal damage the expression of tenascin-C by Müller glia is upregulated, which has been linked to contribute to ECM deposition, retinal mechanical stiffness and a gliotic environment, photoreceptor degeneration and neuroinflammation (40, 85–87). This molecule can interact with other ECM components such as fibronectin and proteoglycans such as aggrecan and neurocan (88), and it has been proposed to promote the generation of stable focal adhesions between Müller glia and the ECM (89), being implicated in the morphology of Müller glia. Interestingly, the effect of tenascin-C on Müller glia mediated neurite outgrowth depends on which Tnc-derived fibronectin type III (TNfn) domains they express, with TNfn domain D being required to promote retinal outgrowth by Müller glia (90, 91). Additionally, tenascin-c has been found to increase Müller glia sensitivity to fibroblast growth factor 2 (FGF2), a factor capable of inducing the dedifferentiation of Müller glia *in vitro*, and tenascin-c knock-out has been reported to impair FGF2 effect on the dedifferentiation state of Müller glia (89).

### 2.2 Remodelling of the ECM

Glial cells are constantly remodelling the ECM through synthesis, degradation, reassembly and chemical modification (92). The main process in ECM remodelling is the cleavage of ECM components, which regulates ECM abundance, composition and structure, and leads to the release of biologically active molecules (e.g., growth factors) that can in turn influence ECM architecture and cell behaviour (93). Different families of proteases can cleave the retinal ECM, the main being matrix metalloproteinase and the adamalysin families.

#### 2.2.1 Matrix metalloproteinases

Matrix metalloproteinases (MMPs) are calcium-dependent endopeptidases that cleave structural motifs of their substrates (e.g., collagens, CSPGs) and have major roles in ECM remodelling, neovascularization and wound healing (94). Depending on their substrate and structural domains, MMPs are classified into: collagenases (MMP-1, −8, −13, −18); gelatinases (MMP-2 and MMP-9); stromelysins (MMP-3, −10, −11); matrilysins (MMP-7, −26); membrane-type matrix metalloproteinases (MT1/2/3/4/5/6-MMP); and unclassified MMPs (MMP-12, −19, −20, −21, −23, −27, −28) (95). Their expression levels in healthy tissue are generally low, and are characterized by an autoinhibitory prodomain that keeps the MMP in an inactive state (96). They are also tightly regulated by the expression of endogenoues tissue inhibitors of metalloproteinases (TIMPs). In the retina, the main MMPs expressed are MMP-2, −3 and-9 (97).

**MMP-2** is the main gelatinase in the retina, where it is constitutively expressed by Müller glia (98) and is activated by the release of MT1-MMP (also known as MMP-14) by RGCs. Different functional studies have identified MMP-2 as a key player in axonal regeneration both via resolution of the glial scar and/or proteolysis of molecular cues that guide axon outgrowth. Indeed, in a retinal progenitor cell (RPC) transplantation study, it was observed that neurite outgrowth was dependent on the activation of MMP-2, which cleaves the CSPG neurocan, thus disrupting its inhibitory effect (99). This was further confirmed in a mouse retinal explant model, where MMP-2 was found to act at the growth cone via a β1-integrin-dependent pathway (100). Furthermore, in a mouse optic nerve crush model, it has been proposed that MMP-2 produced by inflammatory myeloid cells activates Müller glia, which in turn produce anti-inflammatory molecules, such as ciliary neurotrophic factor (CNTF) and interleukin-6 (IL-6), as well as increased levels of MMP-2 that could activate certain intrinsic growth-inducing pathways in the RGCs (101), although the specific mechanisms are yet to be elucidated. MMP-2 is also capable of degrading collagen IV (102), thus altering the integrity of the ILM.

**MMP-3**, mainly expressed by the microglia and in loaded vesicles across Müller glia (97). Its expression is tightly connected to retinal vasculature homeostasis, although its involvement is controversial. While some studies report that MMP-3 contributes to blood-retinal barrier (BRB) disruption and neuroinflammation by degrading tight junction proteins and stimulating the activation of cytokines (103, 104), a more recent study showed that upon inflammation MMP-3 expression at the glia limitans (a barrier formed by the end feet of astrocytes and Müller glia that surround the BRB) is upregulated by Müller glia and promotes the expression of astrocyte-specific tight junction proteins, which in turn tighten the glia limitans reducing leukocyte infiltration and adhesion to the retina (105). Moreover, MMP-3 appears to cleave and inactivate the chemokine CCL2 further restricting leukocyte infiltration.

**MMP-9**, as well as its endogenous inhibitor TIMP-1, is expressed in the nuclei of Müller glia. In healthy retinas, MMP-9 and TIMP-1 form an axis in which TIMP-1 tightly regulates the expression of active MMP-9 (97). MMP-9 primarily cleaves collagen IV and the MMP-9/TIMP-1 axis appears to be key in maintaining normal retinal blood vessel structure and basement membrane integrity (97, 106). However, it has been reported that upon inflammation TIMP-1 tends to aggregate in the cytoplasm of Müller glia, impairing its secretion and reducing TIMP-1 abundance to bind to and inhibit MMP-9 *in vitro* (107). In this context, the overactivation of MMP-9 plays an important role in the promotion of detachment-induced RGC death by excessive degradation of collagen IV, and indeed, its expression has been observed in apoptosing RGCs (97). Furthermore, MMP-9 have been found to cleave both RPE tight junction proteins (108) and blood vessel tight junction protein ZO-2 (106), contributing to the disruption of the BRB integrity and the progression of chronic inflammation. Interestingly, upon photoreceptor damage in a zebrafish model, MMP-9 has been shown to be required for the survival of regenerated cones by modulating the inflammatory response and negatively regulating Müller glia-derived progenitors overproliferation (109).

#### 2.2.2 Adamalysins

Adamalysins are a family of zinc-dependent metallopeptidases conformed by transmembrane-A-disintegrin and metalloproteinases (ADAMs), and secreted A-disintegrin and metalloproteinase with thrombospondin type I motifs (ADAMTSs) that play a key role in ECM remodelling (110).

Regarding membrane bound ADAMs, **ADAM-10** and **ADAM-17** are the best characterized members of this proteinase family due to their ubiquitous expression and involvement in tissue development (111). Both proteinases have been associated with early retinal development via NOTCH1 receptor cleavage, which facilitates neurogenesis by maintaining RPCs in an undifferentiated state, and N-cadherin cleavage, which allows RPC migration and proper retinal lamination (112). Although they are membrane-bound proteases, ADAM-10 and-17 can be released to the ECM by ADAM-15 and-8, respectively, where they are capable of cleaving collagen IV and fibronectin (111). In the developed retina, they are mainly expressed by Müller glia and have been reported to be upregulated in a neovascularization and pro-inflammatory context (113). In the outer limiting membrane (OLM), a tight regulation of ADAM-10 by the Wnt signaling modulator SFRP1, also expressed by Müller glia helps maintain OLM integrity and photoreceptor homeostasis (114). ADAM-10 is also expressed by RGCs and is required for axon guidance and formation of the optic projection (115).

ADAMTSs can be classified by their substrate as: procollagen N-propeptidases, proteoglycanases, fibrillin/fribronectin-associated peptidases and angiogenesis regulators (116). In the retina, Müller glia constitutively express ADAMTS-1, −2, −4, −5, and −13 (117). Especially **ADAMTS-1** and-4, and to a lesser extent also **ADAMTS-5**, have been found to promote synaptogenesis by cleaving the CSPGs neurocan and phosphacan, which inhibit RGCs neurite outgrowth (68, 118, 119), while **ADAMTS-4** thrombospondin repeats may also induce neurite extension independent of CSPG cleavage (120). Meanwhile, **ADAMTS-2** has been reported to cleave fibrillar collagen precursos (type I-III), thus contributing to the maturation and formation of collagen fibers within the ECM (121). As for **ADAMTS-13**, it cleaves the von Willebrand factor, a proteoglycan that participates in platelet aggregation (122); interestingly, while by itself promotes angiogenesis, in the presence of vascular endothelial growth factor (VEGF), a potent vascular permeability and angiogenesis factor, ADAMTS-13 become anti-angiogenic (123).

## 3 Müller glia ECM deposition and retinopathies

It is necessary a balance between the synthesis and degradation of ECM components for tissue homeostasis (92). However, when this equilibrium is disrupted it leads to an abnormal deposition of ECM molecules and changes in the overall stiffness of the tissue. In the retina, several pathological responses cause local changes in the tissue stiffness, exacerbating the process and creating a fibrotic environment.

Fibrosis is a complex biological process that is activated in a tissue after it is wounded. In normal wound healing, myofibroblasts, a cell type key in ECM (mainly collagens) deposition and wound contraction, usually undergo apoptosis once the tissue integrity is restored. However, in fibrotic diseases, myofibroblasts are persistently activated, resulting in the excessive deposition of collagens and severe tissue contraction (i.e., scarring) (124). It has been suggested that ECM stiffness regulates transforming growth factor β (TGFβ)–induced myofibroblast formation in a variety of fibrotic processes in heart, liver, and ocular tissue (125–127), indicating that the increased matrix stiffness caused by the fibrotic process itself promotes myofibroblast formation and further stimulates the fibrotic process. In the retina, the formation of scars has been linked to loss of visual acuity and neurodegeneration (128).

Upon damage, zebrafish Müller glia are capable to re-differentiate into neuronal progenitors, effectively regenerating the damaged retina (129). However, mammalian Müller glia undergo reactive gliosis, characterized by activation, proliferation and hypertrophy. Initially, Müller glia reactivity generate neurotrophic factors to promote the protection of neurons, mainly RGCs (130, 131), but if gliosis persist, they contribute to degeneration and block tissue regeneration (132).

**Figure 3:**
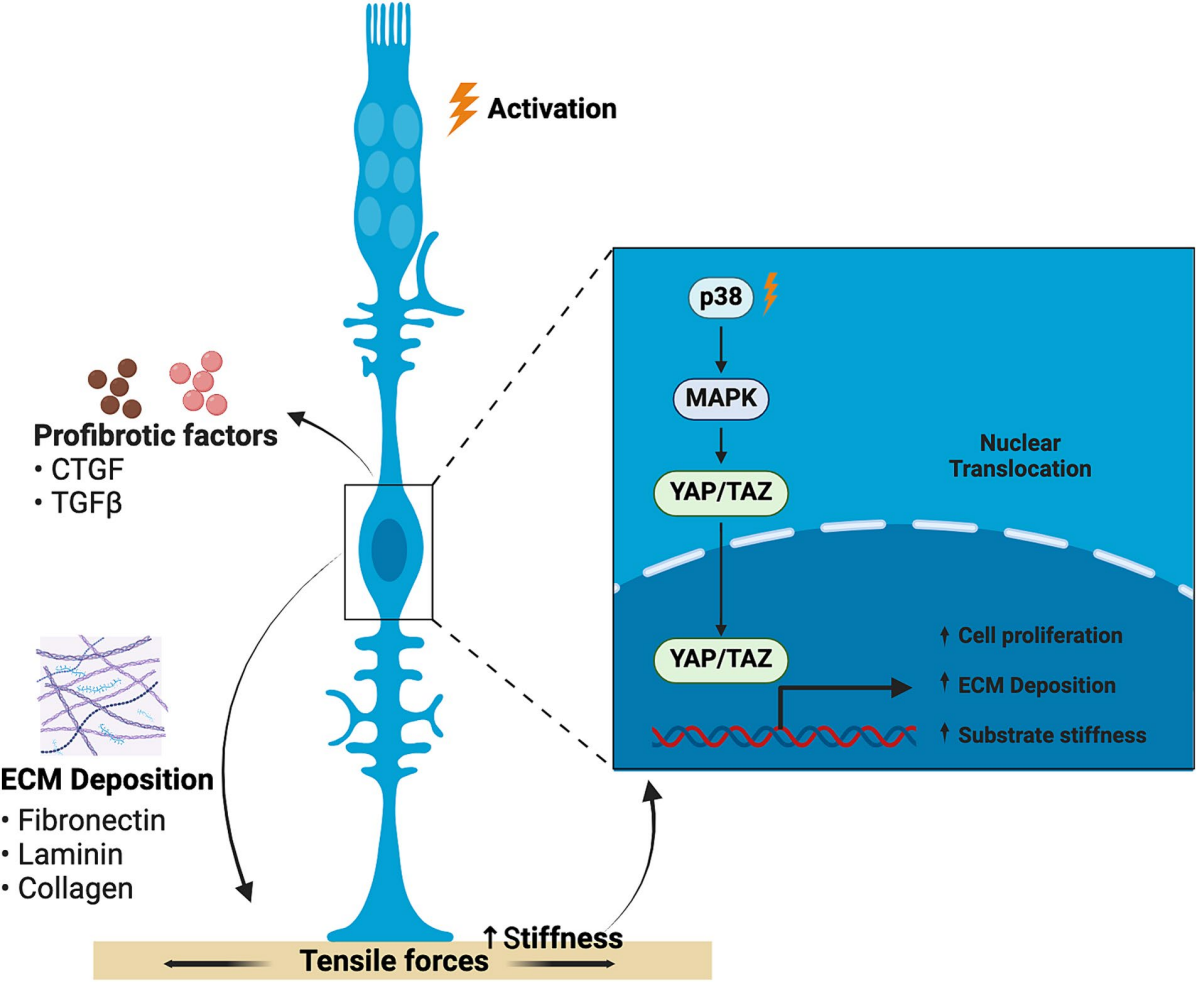
Schematic representation of the feed-forward loop generated by ECM stiffness in retinal fibrosis. Matrix stiffness promotes YAP nucleus translocation and activation, thus increasing cell proliferation and collagen deposition, increasing substrate stiffness in a feed-forward loop. Created with BioRender.com.

The YAP/TAZ complex, a downstream effector of the Hippo signaling pathway that has been shown to affect cellular apoptosis, proliferation and Müller glia reprogramming (133), can be modulated by ECM stiffness (134). A feed-forward loop is settled, in which matrix stiffness promotes YAP translocation to the nucleus and activation, which in turn increases cell proliferation and collagen deposition, thus enhancing substrate stiffness even more (135) (Figure 3). In addition, YAP activation has been linked to TGFβ signaling.

TGFβ signaling is essential to the wound healing process, both in scar formation and tissue-specific regeneration (136). It has been observed that the activation of either canonical or non-canonical TGFβ pathway is associated with the differential injury response in zebrafish and mammals (137). The TGFβ3 isoform, in combination with Smad protein, was the only one upregulated upon damage in zebrafish Müller glia and promoted retinal regeneration via canonical signaling; furthermore, TGFβ3 has been connected to anti-fibrotic wound healing (138). On the other hand, mammal (murine) Müller glia expressed the TGFβ1 and TGFβ2 isoforms, which mediate the non-canonical pathway via p38MAPK (137). Both isoforms promote fibronectin and collagen deposition and stiffening (139, 140). Furthermore, the Notch signaling pathway also promotes Müller glia ECM overexpression working in an additive way with TFGβ, as has been observed both *in vitro* and in an *in vivo* mouse model (141).

Interestingly, the expression of TGFβ1 together with the transcription factor SNAIL has been found to induce the upregulation of several glial-to-mesenchymal transition (GMT) related molecular markers in Müller glia, and the downregulation of glutamine synthetase (142). Also, the interplay between TFGβ and Notch pathways has been linked to the dedifferentiation of Müller glia to an epithelial lineage (143), which correlates to changes in their morphology and their detrimental contribution to retinal fibrosis in chronic gliosis.

**Figure 4:**
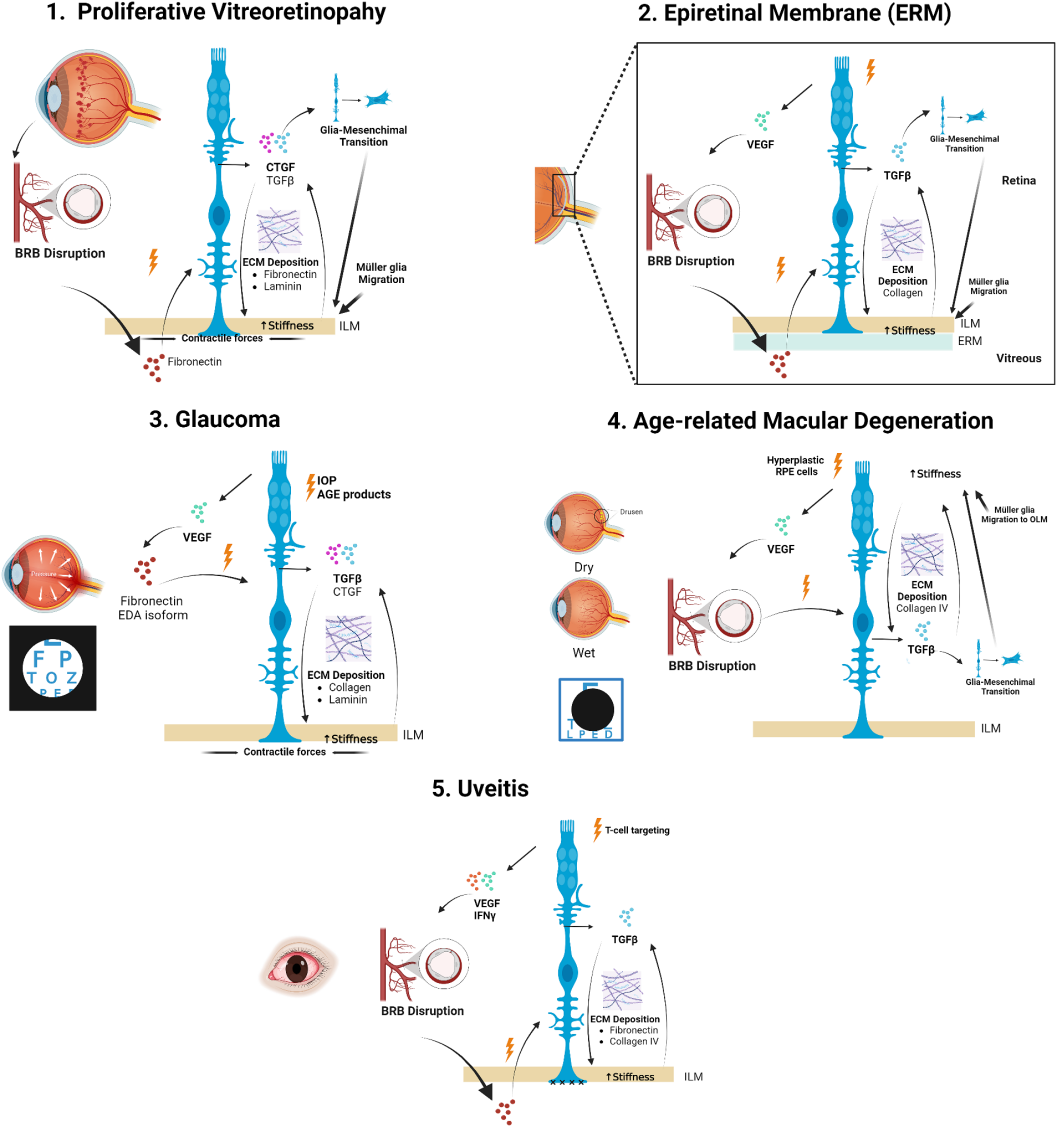
Müller glia-driven alterations in retinal ECM stiffness involvement in retinal diseases. Schematic representation of different retinal pathologies in which ECM deposition and increased stiffness via activated Müller glia is involved and known mechanisms. Created with BioRender.com.

Although fibrosis is a common aspect of different retinopathies, the specific cues driving this process and the ECM components preferentially deregulated are disease-dependent. Therefore, in this section we will discuss the particular mechanisms and ECM components involved in different retinal diseases (Figure 4).

### 3.1 Proliferative vitreoretinopathy

Proliferative vitreoretinopathy (PVR) consists in the formation of fibrocellular membranes on the retinal and posterior hyaloid surfaces following either rhegmatogenous or post-surgery retinal detachment, being the main reason for its failure. The major cell types involved in PVR have a retinal origin (Müller glia and RPE) (144). Although RPE proliferation has been considered for a long time to be the major player in the development of this pathology, in recent years Müller glia have emerged as key in the pathogenesis of PVR (41). Following BRB disruption in PVR, fibronectin in the plasma enters the subretinal space and acts as a chemical attractant, leading to the proliferation and migration of Müller glia into the vitreous, where they synthesize ECM proteins and contract onto the retina, eventually forming a subretinal glial scar (34, 145, 146).

Connective tissue growth factor (CTGF) is thought to be the major mediator of retinal fibrosis in PVR, promoting the synthesis of fibronectin, laminin, and MMP-2 (147), and its expression by Müller glia peaks during late stages of the pathology (148). Müller glia expression of CTGF on substrates of different stiffness was shown to increase on soft substrates (17, 40). This suggests that, when the retina becomes too soft after retinal detachment (since it loses its anchoring points), Müller glia upregulate CTGF in order to stiffen the tissue, which if persisting, leads to PVR.

Apart from synthetizing ECM proteins, in the context of PVR Müller glia themselves develop myofibroblast-like features (141, 149), determined by the expression of smooth-muscle actin. These transdifferentiated myofibroblasts are key in PVR membrane contractility, leading to retinal wrinkling, formation of retinal breaks and reopening of previously sealed ones. They exert this effect on the retina both by contracting themselves and by remodelling the surrounding ECM via TGFβ, contributing to the fibrocellular membrane formation.

### 3.2 Epiretinal membranes

Epiretinal membranes (ERM) are transparent membranes formed at the limit between the vitreous and the retina either as a consequence of diseases (e.g., diabetes, retinal tears), or without any apparent reason – i.e. idiopathic ERMs (iERM) (150, 151). ERMs consist of reactive cellular elements, vitreous structures, and fibrotic components, and can seriously affect vision when developing and retracting in front of the macula (152). Furthermore, the formation and retraction of ERMs increase the rigidity of the retina.

It has been proposed that a main mechanism that leads to ERM formation is the activation of Müller glia by the persisting contact of their processes in the ILM with blood-borne substances that migrate to the vitreous after the disruption of the BRB (18). Therefore, ERMs have been associated with the proliferation of activated Müller glia, which create stronger adhesion between the ERM/ILM complex and the inner retinal tissue, difficulting ERM removal during vitrectomy (153). Furthermore, Müller glia are responsible for newly formed collagens type I, III, and IV in the ERMs, increasing the adhesion to the retina and the stiffness of the ERM and reducing collagen digestibility. Moreover, the upregulation of the intermediate filament GFAP (glial fibrilary acid protein), increases the stiffness of Müller glia, which can also transdifferentiate into myofibroblasts via TGF-β1/2/SNAIL–initiated Müller GMT, contributing to the mechanical properties of ERMs (142, 154). Both collagen deposition and myofibroblast transdifferentiated Müller glia make a rigid and degradation-resistant fibrotic tissue.

The traction exerted by contractile ERM also produce degenerative lamellar holes due to disruption either of the Müller glia cone in the foveola or the connection between the cone and the foveal walls of Müller glia (155). These facts lead to the degeneration of photoreceptors, bipolar cells and horizontal cells in the INL and ONL of the retina.

### 3.3 Glaucoma

Glaucoma is a neurodegenerative disease characterized by the selective death of RGCs, mainly due to an increase in the intraocular pressure (IOP), which leads to a progressive loss of vision, representing the second leading cause of irreversible blindness worldwide (156).

Increased stiffness in the ECM of surrounding tissues such as the trabecular meshwork (TM) has been linked to the progression of glaucoma, where elevated IOP results from increased resistance to aqueous humour outflow as a result of impaired TM cell function (i.e., remodelling of cytoskeleton, increased cell stiffness) and increased ECM deposition (157). Both processes contribute to TM stiffening (158) which affects cell function and increases IOP in a feed-forward cycle. Similarly, in the lamina cribrosa, repeated cycles of increased strain and stiffnening coupled with profibrotic ECM deposition have been associated with glaucoma progression and optic nerve head cupping (159).

In the glaucomatous retina, the enhanced accumulation of advanced glycation end products (AGE) and the expression of their receptor, RAGE, is upregulated, mainly by Müller glia (160). The binding of AGE to Müller glia RAGEs induces the activation of these cells and their release of profibrotic cytokines, such as TGFβ, and an increased expression of ECM proteins (161), increasing retinal stiffness. AGE-binding also induces collagen crosslinking which has been correlated with decreased degradation, loss of tissue compliance and therefore, a higher susceptibility of RGCs to damage (162).

Furthermore, AGE binding to Müller glia have been linked to promoting inflammation and retinal vascularization via the release of VEGF, contributing to the contraction of the fibril matrix and further retinal stiffening (163). VEGF has been associated with the induction of profibrotic growth factors, such as TGFβ1 and CTGF, and ECM components in the early stages of retinopathies (32). Specifically, VEGF may induce the upregulation of cellular fibronectin extra-domain (EDA) isoform, an endogenous ligand of the Toll-like receptor 4 (TLR4) (33). EDA has been shown to be upregulated in glaucoma and contribute to TGFβ2 induced ocular hypertension and ECM deposition (164, 165). AGE accumulation also modifies retinal ECM laminin, which reduces Müller glia Kir4.1 channels expression, thus reducing potassium buffering and leading to RGCs apoptosis (166).

As in other retinal diseases, YAP is heavily involved in the mechanotransduction process. Recently, a genome-wide meta-analysis identified YAP as a potential genetic glaucoma risk factor (167); therefore YAP may play a prominent role in glaucoma pathogenesis, driving the establishment of Müller glia TGFβ activation-retinal stiffening feed-forward loop (135).

### 3.4 Age-related macular degeneration

Age-related macular degeneration (AMD) is a progressive, multi-factorial neurodegenerative disease that is the leading cause of vision loss in western elderly population (168). AMD primarily affects the macula, a small structure within the central retina containing cells of the neuroretina (neurons and glial cells), and RPE attached to the Bruch’s membrane (a pentalaminar ECM that connects the RPE to the choriocapillaris) (169). The macula has the highest density of photoreceptors in the retina, and photoreceptor degeneration is one of the main consequences of AMD (170).

Stress-relaxation studies of retinal explants have shown that AMD retinas are stiffer than healthy ones (171), while genome-wide association studies (GWAS) linked a dysregulation of the collagen IV ECM pathway to the pathogenesis of AMD (172). Early stages of AMD are characterized by the accumulation of drusen, extracellular deposits of protein and lipid aggregates, which appear as yellowish dots (173). More advanced forms of the disease include the “dry” or atrophic form characterized by the progressive dysfunction of the RPE, that anchors the outer retina, and underlying choriocapillaris resulting in loss of photoreceptors and retinal degeneration. “Wet” or neovascular form, less frequent, is characterized by choroidal neovascularization (CNV) that produce leakage into the retina, Müller glia activation and RPE detachments (169, 174, 175).

In CNV, hyperplastic RPE cells, i.e., their proliferation is abnormally upregulated, and the vascular leak and subsequent development of subretinal fibrosis leads to the disruption of Müller glia, which activate the TGFβ1/Smad3 signaling pathway, associated to the stiffening of the matrix (176). Additionaly, endoglin (a co-receptor for the TGFβ family) is strongly expressed on fibro-neovascular tissue and hyperplastic RPE cells, promoting fibro-neovascularization through Müller–derived VEGF and could be also involved in Müller glia GMT.

Interestingly, upon photoreceptor loss Müller glia both extend their processes and proliferate beyond the outer limiting membrane (OLM) creating a dense gliotic membrane and “sealing the gaps” in atrophic areas (177). This Müller glia migration is stimulated by the accumulation of drusen attached to the choroid and their interaction with the RPE resulting in the production of tumour necrosis factor α (TNFα), TGFβ, and VEGF by both cell types, further driving photoreceptor degeneration.

GWAS analysis also identified a highly penetrant missense rare variant in the coagulation factor II thrombin receptor-like 2 (F2RL2), expressed by Müller glia of human and pig retinas (172). F2RL2 has been associated to fibrotic, neovascularized areas in advanced AMD, suggesting a role in the progression of the disease.

### 3.5 Uveitis

Uveitis comprises a diverse group of intraocular inflammatory diseases of both the uvea (i.e., the iris, ciliary body, and choroid) but also adjacent tissues including the retina. Uveitis is mostly idiopathic but could also developed as a result of infectious and autoimmune processes (178).

In the retina, uveitis mostly displays an autoimmune aetiology, where CD4+ T-cells recurrently target the tissue, as a result of the breakdown of the BRB. In this context, Müller glia have been shown to contribute to the disease by becoming gliotic and upregulating the expression of pro-inflammatory cytokine interferon gamma (IFNγ) and downregulating the neurotrophic factor pigment epithelium-derived protein (PEDF), which under physiologic conditions counteracts the activity of angiogenesis inducers (179). Furthermore, they also contribute to BRB breakdown by releasing VEGF (180).

Uveitis has been associated with the disintegration of the ILM due to the disruption of the fibronectin matrix. Müller glia upregulate fibronectin, which changes its expression pattern from a continuous band to a spotted one. This change, in addition to alterations to Müller glia morphology consequence of the gliotic process, leads to the loss of Müller glia end-feet connection at the ILM, and thus, its disruption (72).

In addition, in infection-derived uveitis the p38MAPK pathway is activated in Müller glia, which has been associated to the expression of pro-inflammatory factors (e.g., IL-6, ICAM-1, CXCL1, CXCL10, CCL2 and CCL5 and CCL7), ECM deposition (mainly fibronectin and collagen IV) and tissue stiffening (137, 181).

## 4 Modelling the retinal ECM

Although *in vitro* research cannot fully recapitulate *in vivo* conditions, it presents many advantages over *in vivo* studies. Mainly, it allows a tight control of the chemico-physical environment, and displays higher throughput, reduced cost and restricted animal-use.

Traditionally, cell cultures are performed on materials such as glass and polystyrene (plastic). However, the stiffness of these materials is in the range of gigapascals (GPa) while the retinal stiffness ranges between 1.3 to 25.9 kPa (182). Therefore, the cellular behaviour observed on glass/plastic might not correlate with what is actually happening *in vivo*, where the ECM comprises a complex, changing milieu. Nonetheless, *in vitro* studies of Müller glia grown on substrates displaying physiological stiffnesses are somewhat lacking. Thus, in this section we will discuss biomaterial alternatives to culture Müller glia.

In recent years, different substrates have been developed aiming to bridge the gap between *in vitro* and *in vivo* models. Biomaterials allow controlling their mechanical, compositional, and structural properties, thus more closely resembling the native tissue. They not only provide more physiological culture conditions, but could also be employed in new therapeutic approaches focusing on the mechanical environment of the retina. Among the different biomaterial systems developed, hydrogels—aqueous-swollen crosslinked polymeric networks —have emerged as the most promising option for cell culture. By varying the hydrogel composition and the gelation conditions it is possible to tune their biophysical and biochemical properties (stiffness, pore size, fiber alignment), enabling them to support cell adhesion and protein sequestration (183–185). Hydrogels are derived either from natural or synthetic materials, providing a different set of advantages and disadvantages that will be explored.

### 4.1 Natural hydrogels

Natural materials for hydrogel fabrication are mostly isolated from native ECM. Due to their origin, they present many similarities to the physiological environment. Furthermore, they contain adhesive sites for cell attachment, not requiring additional functionalization nor surface modifications (186) (Table 2).

**Table 2:** Natural biomaterial candidates for retinal ECM modelling *in vitro.*

Material	Origin	Advantages	Disadvantages	Neuronal support	References
Collagen	Rat tail tendon	Biocompatible Biodegradable Promote cell adhesion, proliferation and motility	Limited long-term stability Batch-to-batch variability Low stiffness; requires extensive crosslinking to tune	Fail to induce neuritogenesis	(187, 191, 196)
Matrigel	Engelbreth-Holm-Swarm mouse sarcoma tumours	Reconstituted basal membrane Maintains stem cell self-renewal and pluripotency	Not well-defined Batch-to-batch variability Xenogenic contaminants Low stiffness	Compliance to culture and expand retinal organoids; Müller glia and RGC-like cells obtained in Matrigel	(198, 199, 203, 205)
Alginate	Brown algae	Biocompatibility Low citotoxicity	For mammalian cell culture it needs peptide coupling Poor long-term stability Batch-to-batch variability Low mechanical integrity	Compatibility with CNS tissue Promotes outgrowth of regenerating axons Promotes differentiation to retinal progenitor cells	(210, 211, 213, 216)

#### 4.1.1 Collagen hydrogels

Collagen is the main component of the ECM of most soft tissues. Thanks to its natural origin, collagen gels are biocompatible and biodegradable and present low cytotoxicity, while promoting cell adhesion, proliferation and motility (187) via α_1_β_1_ and α_2_β_1_ integrin-binding sites (188). Furthermore, collagen hydrogels are highly porous allowing free diffusion of ions and molecules to support cell growth through the polymer matrix (189).

Since collagen type I comprises 90% of fibrillar collagens (190), it is used preferably to prepare hydrogels, although collagen type IV can also be used. Collagen used as polymer material is usually derived from solubilized type I collagen obtained from rat tail tendon and gelation of these solutions is achieved by raising the temperature and the pH to initiate collagen fibril self-assembly (191). Interestingly collagen hydrogel stiffness can be tuned by increasing the pH (above pH 10.0) while decreasing polymerization temperature; this changes augment fibril packaging thus increasing overall matrix stiffness (192, 193).

Collagen hydrogels have been sparsely used for culturing retinal cells such as RPE cells in an AMD model (194), while their use in Müller glia culture has been mostly restricted to contractility essays (195). In cultures of cerebellar granule neuron-glial spheroids, collagen type I, but not type IV, hydrogels have been shown to stimulate astrocyte proliferation to a certain extent but fail to induce neuritogenesis and Calcium signaling activity (196), which suggest that collagen hydrogels might be a poor candidate for CNS tissue mimicking and thus to culture retinal cells. Indeed, previous studies in which the influence of ECM in the neurite branching and regrowth of RGCs was addressed and only a discrete effect was found (73).

In general, collagen hydrogels present some important drawbacks including limited long-term stability and batch-to-batch variability. These hydrogels display low stiffness, requiring extensive chemical crosslinking to achieve stiffnesses higher than 1 kPa (191). This crosslinking leads to significant matrix contraction and altered degradability, restricting the culturing time. Furthermore, cells cultured may alter the intrinsic mechanical properties of the hydrogel (197).

#### 4.1.2 Matrigel

Matrigel is a commercial preparation of ECM components obtained from Engelbreth–Holm–Swarm (EHS) mouse sarcoma tumours; it is composed primarily of laminin, collagen type IV, and entactin, with various other constituents including proteoglycans, such as perlecan, metalloproteinases and growth factors (including the TGFβ family) (198). Matrigel is considered a reconstituted basement membrane that undergoes gelation at temperatures in the range 22–37°C, when entactin acts as a crosslinker between the laminin and collagen IV to create the hydrogel (199).

Matrigel ability to maintain self-renewal and pluripotency due to its tumorigenic origin makes it a good candidate for culturing stem cells. Indeed, as a thin gel coating, Matrigel has been used to culture and expand human pluripotent stem cells (hPSCs) (200) and neuronal stem cells (201), while thicker coatings have been used to investigate angiogenesis (202).

Matrigel has been used as substrate to develop retinal organoids, where different groups have achieved the differentiation of human induced Pluripotent Stem Cells into Müller glia and further expand the obtained cells; these cells were functional and expressed typical Müller glia markers (203, 204), which suggested that Matrigel’s enriched ECM composition favoured Müller glia differentiation. Likewise, Matrigel was also used to differentiate mouse Embryonic Stem Cells into RGC-like cells (205) and human Embryonic Stem Cells into RGCs and Müller glia precursors (206).

Due to its origin, Matrigel is not well-defined and present high batch-to-batch variability and xenogenic contaminants (191, 199), which leads to a significant level of uncertainty to experimental results. Furthermore, Matrigel stiffness is relatively low (around 400 Pa) and due to its temperature sensitivity drops to lower stiffnesses if it is not kept at 37°C during experimentation (207); although it can be tuned by increasing overall protein concentration, this leads to alterations in its biological functionality.

#### 4.1.3 Alginate

Alginate is a polysaccharide typically obtained from brown algae that presents biocompatibility and low toxicity for biomedical applications. It is formed by α-L-guluronate (G) and β-D-mannuronate (M) residues, and hydrogels are formed by the interaction of G residues with different divalent cations (mainly calcium and barium) (11).

The stiffness of the gel depends on the overall G section (208) and the cation used for gelation (209). Since mammalian cells lack receptors for alginate, and alginate gels show low protein adsorption, their coupling to specific peptides is required for proper cellular adhesion (210). The most commonly used peptide is arginine-glycine-aspartic acid (RGD), which concentration required for cell adhesion and growth is cell-type dependent.

RGD-alginate gels have been found to display compatibility with CNS tissue, promoting the outgrowth of regenerating axons and the elongation of astrocytic processes of the spinal cord (211) and favouring the expansion of hipoccampus neural progenitor cells *in vitro* (212). These hydrogels have also been shown to promote the generation of retinal progenitor cells from iPSC-and hESC-derived embryoid bodies (213). Although there are no studies of Müller glia grown on alginate hydrogels, this biomaterial has been used in brain astrocyte activation studies where different alginate concentrations elicited a different stiffness-mediated response (214, 215); as Müller glia take up the mechanosensor function in the retina that astrocyte carry out in the brain and both cell types are glial cells of the CNS these results suggest that alginate hydrogels could be a good alternative to study the effect of substrate stiffness on Müller glia.

However, as in other natural hydrogels, alginate shows poor long-term stability, and batch-to-batch variability in their degradation and mechanical properties (216). Furthermore, native alginate gels display low mechanical integrity, as a result of their swelling behaviour in the aqueous culture environment, requiring the incorporation of a compatible reinforcing agent (e.g., cellulose, polylactic acid) within the alginate structure (217).

### 4.2 Synthetic hydrogels

As opposed to natural hydrogels, synthetic hydrogels are well-defined and their chemical–physical properties can be easily tuned (218), which may be more suitable for cell culture and native ECM modelling, although they do not have any inherent bioactivity (Table 3).

**Table 3:** Synthetic biomaterial candidates for retinal ECM modelling *in vitro.*

Material	Origin	Advantages	Disadvantages	Neuronal support	References
Polyacrylamide	Mixture of acrylamide and bis-acrylamide	High reproducibility Strong mechanical homogeneity Citocompatibility Independent tunable stiffness	Bioinert, needs to be functionalized with ECM components	Soft hydrogels promote neural extension	(40, 219, 222, 226, 227)
PDMS	Mixture of an elastomer base (PDMS oligomers, a platinum catalyst and silica) and a curing agent	Biocompatibility High oxygen permeability Thermal stability High protein binding capability Tunable mechanical properties	Bioinert, needs to be functionalized with ECM components	CNS compliant Promotes the differentiation of different retinal cells, including RGCs, in retinal organoids	(11, 230, 231, 236, 238, 239, 241)
PEG	Polymer of ethylene oxide that display a variety of crosslinked end groups	Biocompatibility Low citotoxicity Hydrophilic; good metabolite diffussion	Bioinert, requires functionalization by ECM protein Limited amount of protein that can be coupled to the gel substrate that can lead to weak adhesions Stiffness cannot be independently tuned	Expansion of neural progenitor cell cultures and differentiation to glial cells Retinal organoid culturing	(238, 242, 243, 246, 248, 250)

#### 4.2.1 Polyacrylamide gels

Polyacrylamide is a cyto-compatible, bioinert material, formed by the synthetic polymer acrylamide and its crosslinker (bis-acrylamide), gelation being activated by ammonium persulfate (APS) and tetramethylethylenediamine (TEMED). Polyacrylamide gels exhibit a strong homogeneity in surface topography, mechanical properties, and coating density, displaying high reproducibility (219).

Polyacrylamide hydrogels stiffness can be easily tuned over several orders of magnitude within the physiological range, by varying the concentration of the constituents (219, 220). Although the gel elastic modulus increases with increasing cross-link concentration, the formation of highly cross-linked clusters add heterogeneity to the network structure, leading to an inflection point, after which gel stiffness decreases under high cross-linking conditions (221). Therefore, crosslinking should be tightly controlled when fabricating the hydrogel. Due to their bioinert characteristics, polyacrylamide hydrogels need to be functionalised by the tethering of ECM components in order to support cell adhesion, including poly-L-lysine (222), laminin (14) or collagen I (223), among others. This tethering is UV-dependent.

One of the major disadvantages observed in polyacrylamide gels is the change in porosity dependent on the gel formulation (224). However, Wen et al. (225) have shown that porosity and stiffness can be independently tuned. Furthermore, when they cultured mesenchymal stem cells on polyacrylamide gels of different stiffnesses and porosities they observed that differentiation into either adipocytes or osteocytes did not depend on porosity or protein tethering but only on the substrate stiffness, which makes polyacrylamide gels good candidates for studying substrate stiffness as an isolated parameter.

Polyacrylamide gels are ideal for CNS mimicking. Cultures of adult neural stem cells on soft polyacrylamide gels promote neural expansion while stiffer ones favoured the appearance of glial cells of astrocytic phenotype (226, 227). Likewise, the survival and proliferation of oligodendrocyte progenitor cells has been shown to be modulated by substrate stiffness (228).

Thanks to polyacrylamide capacity to tune its rigidity over a wide range of stiffnesses, they can be used to model pathologic contexts, where stiffness is heightened. For example, a stiff polyacrylamide matrix led to impaired myosin activity and branching inhibition in an oligodendrocyte differentiation study (229).

In the study of retinal fibrotic diseases, Müller glia cultured on polyacrylamide gels showed that on stiff substrates there is an upregulation of stress fibers and reorganization of the cytoeskeletal integrity, changes in gene regulation and a morphologic transition to a myofibroblast-like phenotype (39, 40). This is due to stiffening-stimulated YAP activation by the TGFβ1-PI3K/Akt pathway (15) and the expression of CTFG, implicated in the gliotic process (17).

Polyacrylamide gels manufacturing parameters such as UV activation intensity and exposure time, gel thickness, and acrylamide/bis-acrylamide concentration should be tightly controlled to achieve accurate, reproducible results (219).

#### 4.2.2 Polydimethylsiloxane gels

Polydimethylsiloxane (PDMS) is a silicon-containing polymeric material. PDMS hydrogels are formed by an elastomer base (PDMS oligomers with vinyl-terminated groups, a platinum catalyst and dimethylvinylated/trimethylated silica) and a curing agent (dimethyl methylhydrogen siloxane and tetramethyl tetravinyl cyclotetrasiloxane) (11). PDMS hydrogels display biocompatibility, high oxygen permeability, thermal stability, a high protein binding capability for cell expansion and tunable mechanical properties (230, 231). Substrate stiffness can be easily tuned by curing temperature and time and changing the base-to-curing agent ratio (232), where an increased ratio correlates to augmented stiffness.

The elastic modulus of PDMS is typically in the order of megapascals (233). However different strategies, i.e., altering the geometrical organization by fabricating microposts of different heights (234), have been developed to decrease the effective stiffness of PDMS hydrogels, making them apt for mimicking soft tissues such as the CNS.

PDMS hydrogels have also been used to study mechanotransduction in different glial cells of the CNS. Thanks to their elastic properties they have been applied as a stretchable material to test the effects of tensile forces on oligodendrocyte progenitor cells (235), which have been shown to favour cell expansion and differentiation. In other studies, tuning their rigidity have been used to study the effect of mechanical stiffness on cortical astrocyte activation and progression of astrogliosis (236), which could be extrapolated to Müller glia. Also for Schwann cell proliferation and transcription of basal lamina receptor genes in axonal sorting and myelination which is promoted by the substrate stiffness activation of the YAP/TAZ complex (237). Likewise, PDMS have been used as substrate for the developing of retinal organoids, where mESCs were differentiated mainly into photoreceptors, but also RGCs, amacrine cells, horizontal cells, bipolar cells and Müller glia (238, 239).

However, PDMS being a synthetic material, is bioinert and require the adsorption of charge enhancers, e.g., poly-L-lysine (236), and/or the binding of adhesive ECM proteins, e.g., laminin, fibronectin (240, 241), for proper cell attachment and integrin signaling. Thus, the nature of the generated interactions and the coating density are critical for cell adhesion and spreading.

PDMS hydrogels are preferentially used when looking for larger structural stiffness, while in starting studies of compliant substrates polyacrylamide gels are usually chosen (219).

#### 4.2.3 Polyethylene glycol gels

Polyethylene glycol (PEG) is a hydrophilic polymer of ethylene oxide that display a variety of end groups (e.g., alcohol, methyl ether, amine, N-hydroxysuccinimidyl (NHS) ester) which are cross-linked for hydrogel formation (242).

PEG is hydrophilic, present biocompatibility and low toxicity, is non-adhesive toward proteins and cells and its stiffness can be tuned by varying the polymer concentration. As with other synthetic hydrogels PEG is bioinert and as such requires functionalization by ECM protein (e.g., vitronectin, fibronectin) binding for cell attachment (243–245). PEG hydrogels can mimic soft substrates; however, softer gels (~1 kPa) are somewhat limited in the amount of protein that can be coupled to the gel substrate, which can pose a problem for cells with weak adhesion forces (246). Alternatively, PEG cross-linking to natural-based materials, such as gelatin, and natural-based gelatin cross-linking has been shown to enhance the biophysical properties, including gelation time, and biocompatibility of PEG hydrogels improving cell long-term viability (247).

Although less frequently than polyacrylamide or PDMS gels, PEG hydrogels have been utilized to study neural cell biomechanical behaviour, increasing PEG composition and consequential increase in stiffness has been associated with the expansion of neural progenitor cell cultures and differentiation to glial cells (248). PEG hydrogels have also been fabricated for retinal organoid culturing, their intrinsic inertness and hydrophilicity allowing for a good diffusion of metabolites as well as factors, favouring organoid development and colony formation in this type of essay (238). Although PEG hydrogels (fused with hyaluronan acid) have been proved to be non-citotoxic to Müller glia *in vitro* (249), no further studies have been carried out.

Interestingly, RGCs grown on PEG hydrogels of different stiffnesses, coupled with poly-L-lysine, attached better in hydrogels with an stiffness range of 3.8–5.7 kPa than on expected optimal stiffness hydrogels (0.9–1.8 kPa), due to the ratio of free amines to hydroxyls (250), which suggests that stiffness cannot be measured as an isolated parameter in PEG hydrogels.

### 4.3 Two-dimensional vs. three-dimensional culture

Traditionally, cell cultures have been performed on two-dimensional (2D) conditions. Although these approaches have significantly advanced our understanding of cell behaviour, cell bioactivity on 2D systems not always correlates with what happens *in vivo*, where cells are embedded in the three-dimensional (3D) native ECM. In fact, cell encapsulation in 3D systems demonstrate that increasing the dimensionality can impact cell proliferation, differentiation, mechanoresponse, and cell survival, making them good candidates for tissue-engineering (251, 252).

Cells grown in 2D monolayers rely on their adherence to a flat surface and have unrestricted access to nutrients and growth factors present in the culture medium, which results in homogenous growth and proliferation (253). This allows for simple and efficient cell culturing, but lacks important mechanical cues since cells *in vivo* receive stiffness signals from all over their surface. Additionally, cells display forced apical-basal polarity that does not occur *in vivo* (254). Meanwhile, 3D cultures allow the formation of more cell–cell and cell-matrix interactions that more closely resembles native tissues, but could also display oxygen and nutrient restrictions, reducing their viability (255).

In retinal cells, a study in which perinatal Müller glia were investigated for neuron cell replacement therapy, culture in 2D conditions and encapsulating spheroids showed differential neuronal gene expression (256). For example, expression of *Mdk* and *Sox3*, regulators of neurogenesis, was upregulated only in 2D cultures. In contrast, *Mef2c*, involved in early neuronal differentiation, was robustly expressed only in 3D conditions. They also observed some genes associated with dendrite formation, neurite outgrowth and synaptic refinement, such as *Bmp2*, *Ptn* and *Nptx1*, upregulated only in 3D conditions.

Regarding the production of retinal organoids, although different groups have been able to successfully differentiate iPSCs or ESCs into different retinal cell types (213, 238, 257), the small size and the heterogeneity found pose significant technical problems such as lower sensitivity or the difficulty to distinguish the origin of the measured signals.

Assessing the mechanical response of cells to substrate stiffness is significantly easier in 2D cultures (258), where cells adhere their basal surface along a planar surface and are able to detect stiffness over the length of a single cell (259). However, in 3D systems, the whole cell is in contact with other cells and/or the ECM. Furthermore, ECM stiffness can vary depending on the ECM ligand density and pore size, and intra-and extra-fibril crosslinking and alignment. Therefore, it is possible for an individual cell to locally sense part of a matrix as stiff or soft depending on whether tension is generated parallel or perpendicular to a particular fiber. Regarding the materials employed, hydrogels used for 3D culture have shown to facilitate matrix production/degradation and other cell bioactivities, that highly affect matrix stiffness and viscoelastic properties, which has been deemed complex to control and isolate (254).

While 3D cultures better recapitulate *in vivo* overall ECM conditions, their complexity difficult the independent study of mechanical parameters, such as ECM stiffness. Conversely, in 2D cultures the effect of these parameters can be effectively isolated from one another.

## 5 Conclusions and future directions

The retina, due to its location, is constantly exposed to mechanical changes. Furthermore, the tissue itself is very heterogeneous, displaying cells with different stiffnesses and ECM layer-dependent composition. The mechanical state of the retina, and specifically its resistance of deformation or stiffness, is key in its development and maturation, and aberrant ECM deposition and increased stiffness is associated with the progression of different retinal diseases, including retinal fibrosis, PVR, glaucoma, AMD, uveitis and the formation of ERMs.

Müller glia, as the main retinal glial cell type, are capable of both sensing mechanical changes in the whole retinal thickness and deposit ECM constituents. During retinal diseases, Müller glia become gliotic and hypertrophic, promoting a tissue stiffening feed-forward loop by secreting profibrotic and proinflammatory factors and ECM components, and transitioning to a myofibroblast state.

The interplay between retinal stiffness and the biological response of the mechanosensitive Müller glia is a research area of growing interest. As more sophisticated *in vitro* models are being developed, this would lead to new findings into the effect of substrate stiffness in retinal physiology and disease, and promote the elucidation of novel diagnostic targets. To better mimic the effect of matrix stiffness *in vitro*, 2D cultures in synthetic materials appear to be ideal candidates for retinal ECM modelling. However, most Müller glia and other retinal cells *in vitro* studies where these biomaterials have been used were focused on retinal organoid differentiation and expansion. So far, Müller glia *in vitro* studies focusing on the stiffness of the substrate have been mainly been done on polyacrylamide hydrogels, which display high biocompatibility and stability, low toxicity and are bioinert. Additionally, their stiffness can be tuned over a wide physiological range and allow for the study of the substrate stiffness effect on Müller glia behaviour independent of other mechanical parameters. Nonetheless the culture of other glial cell types in different biomaterials show promising results that could be extrapolated to Müller glia. To more closely resemble the native ECM complexity, in future, hydrogel 3D cultures would be a better option, but oxygen and nutrient restrictions as well as effectively decoupling the stiffness from other mechanical parameters need to be overcome first.

This opens up a field of mechanical therapeutic approaches to retinal disease focusing on the modulation of the ECM stiffness via Müller glia to tackle the fibrotic component present in many of these pathologies. Furthermore, improved, more native-like hydrogels could help advance the field of cell transplant therapies by providing a compliant environment for the cells to grow and differentiate. With this review, we aim to emphasize the role of Müller glia in the ECM stiffness of the retina and its importance in retinal homeostasis, and offer an insight into different hydrogel systems to model the ECM stiffness *in vitro*, which has great research value and translational potential in the treatment of retinal diseases.
